# Is Chagas Disease Really the “New HIV/AIDS of the Americas”?

**DOI:** 10.1371/journal.pntd.0001861

**Published:** 2012-10-25

**Authors:** Rick L. Tarleton, James W. Curran

**Affiliations:** 1 Center for Tropical and Emerging Global Diseases, University of Georgia, Athens, Georgia, United States of America; 2 Rollins School of Public Health and Emory Center for AIDS Research, Emory University, Atlanta, Georgia, United States of America; Yale School of Public Health, United States of America

Chagas disease has rightfully earned the designation as “the most neglected of the neglected diseases” [1]. Most of the 10–20 million people already infected with *Trypanosoma cruzi*, the protozoal agent of Chagas disease, are untreated, despite the existence of effective therapies. Diagnostics are largely archaic (xenodiagnosis—using the blood-feeding insect vectors to detect infection—is still in use), vaccines are non-existent, and prevention is predominantly based upon periodic, expensive, and laborious insecticidal spraying of houses to deter infestation by the insect vectors.

Like other neglected tropical diseases (NTDs), Chagas disease is primarily a disease of poverty, disproportionately affecting the rural poor and immigrant populations predominantly in developing countries. The even greater “neglect” of Chagas disease relative to other NTDs is more difficult to explain; perhaps the fact that Chagas disease is not a truly global problem and especially its absence from Africa, where both NTDs and the “big three” (HIV, malaria, and tuberculosis) have their most devastating impact, contributes to its relative obscurity. Additionally the rather modest impact of Chagas disease in developed countries, where HIV and tuberculosis continue to pose considerable threats, is a factor. This inattention to Chagas disease is reflected in and likely perpetuated by the abominable state of research and operational funding for Chagas disease [2].

Given this obscurity, we are appreciative of the attempts of Hotez et al. to bring attention to Chagas disease in their May 2012 editorial in *PLOS NTDs* [3]. Unfortunately, casting Chagas disease as “the new HIV/AIDS of the Americas” is a forced and inaccurate comparison. The lay press has flamed this fire with more sensationalized stories with headlines such as “America hit by new ‘aids’ called Chagas” (Weekly Blitz), “Increased Incidence of Chagas Diseases Alarms US Health Officials” (Press TV), “Chagas Disease, an Incurable Infection, Called the ‘New AIDS of the Americas’” (Canada Free Press), “Chagas Disease: A New Global Pandemic?” (Fox News), and “New ‘AIDS’ Chagas Discovered, Ravages Americas • Over 8 million Infected” (Nigerian Tribune).

HIV and *T. cruzi* are both infectious agents that cause life-long infections and, like all blood-borne pathogens, are potentially transmitted by blood transfusion and congenitally from mother to newborn. That is essentially the extent of similarity between these two infections. Other than as noted above, modes of transmission of HIV (predominantly sexual) and *T. cruzi* (vector-borne) are quite different. HIV infection is also nearly always fatal unless treated, and continuous treatment has to be maintained for life. In contrast, the majority of individuals infected with *T. cruzi* controls the infection without treatment and show no overt clinical symptoms associated with Chagas disease.

Although not a uniform death sentence, *T. cruzi* infection is far from innocuous, as an estimated 30%–40% of infected individuals develop debilitating and chronic disease, and accounts for 20,000–50,000 deaths per year. There are several effective drugs for treating Chagas disease, and the course of treatment is substantial (30–60 days), but not life-long. It is often stated, and is reiterated in the Hotez editorial [3], that the available treatments are only effective if given early during *T. cruzi* infection. This is not true; drug treatment has proven effective in curing the infection and in preventing the progression of disease when given at any point in the infection [4]–[8]. Unfortunately, benznidazole and nifurtimox, the currently available therapeutics for *T. cruzi* infection, are not uniformly efficacious and both have significant side effects that result in a sizable number of individuals not completing a full course of treatment [9]. However, the greatest problem associated with drug therapies for Chagas disease is in simply determining if the treatment has successfully cleared the infection—for *T. cruzi* infection, this is particularly difficult to do. Thus, the high relative toxicity, long course of therapy, and its uncertain success all combine to make these current drug treatments grossly underutilized. The cost of treatment, at least at the patient level, is less of a factor in many countries, including Argentina, Brazil, Venezuela, Colombia, and Ecuador, where the government health systems bear these expenses.

Unlike HIV, *T. cruzi* has infected humans and other mammals in the Americas, including the United States, for thousands of years [10]. Because the natural transmission cycle for *T. cruzi* already exists in the United States and the fact that transmission to humans is quite inefficient, it is highly unlikely that the immigration of *T. cruzi*–infected people or animals into the United States will lead to increased/uncontrolled transmission in the United States. The transmission dynamics of *T. cruzi* (except as noted above, person-to-person spread is essentially absent) also makes it improbable that the migration of infected hosts to “non-endemic” areas will establish transmission foci. So Chagas disease, while a serious public health problem, has little or no danger of explosive epidemic spread.

In short, while the comparison of Chagas to HIV/AIDS is forced and misleading, Chagas disease deserves much more international attention in its own right. Chagas disease *is* a major health problem of the Americas but should be a totally manageable problem, given the right resources and reasonable plans [8], [11], [12]. Starting points in the process are the development and implementation of better diagnostics, so that the millions with *T. cruzi* infection can be identified and treated before they suffer irreparable disease or before they pass on the infection to others, in particular newborns. Development of safer and more effective drugs is also crucial. Fortunately, academic, corporate, and public-private partnerships are all collaborating to help fill the drug development pipeline [13]. Assessing treatment efficacy is also a major challenge for the development of new and better drugs, so the discovery of biomarkers of therapeutic efficacy has to be a very high priority. Vector control and housing improvements will likely remain key components in preventing the further spread of infection in endemic areas. It is encouraging that the sequencing of the first *T. cruzi* vector genome (of *Rhodnius prolixus*) has recently been completed and will hopefully reveal new targets for vector control and that novel methods for this control are being put into use[14], [15]. And despite extremely limited resources, the Chagas disease community has much to look forward to that is positive, including the results of a number of clinical trials of new drugs that are currently just getting underway.

A “call to action” for Chagas is certainly justified, but unrealistic claims and analogies are not helpful. In the history of HIV/AIDS, advocacy has been—and remains—critically important but it was neither effective nor sustainable when it conflicted with the hard realities of science and epidemiology.
